# CSIG promotes hepatocellular carcinoma proliferation by activating c-MYC expression

**DOI:** 10.18632/oncotarget.2900

**Published:** 2015-02-28

**Authors:** Qian Cheng, Fuwen Yuan, Fengmin Lu, Bo Zhang, Tianda Chen, Xiangmei Chen, Yuan Cheng, Na Li, Liwei Ma, Tanjun Tong

**Affiliations:** ^1^ The Peking University Research Center on Aging, Department of Biochemistry and Molecular Biology, Peking University Health Science Center, Beijing, China; ^2^ Department of Microbiology, Peking University Health Science Center, Beijing, China; ^3^ Department of Pathology, Peking University Health Science Center, Beijing, China; ^4^ Department of Histology and Embryology, Peking University Health Science Center, Beijing, China

**Keywords:** CSIG, HCC, MYC, proliferation, protein degradation

## Abstract

Cellular senescence-inhibited gene (CSIG) protein significantly prolongs the progression of replicative senescence, but its role in tumorigenesis is unclear. To reveal the role of CSIG in HCC, we determined its expression in HCC tissues and surrounding tissues and its functions in tumor cell proliferation *in vitro* and *in vivo*. CSIG protein was overexpressed in 86.4% of the human HCC cancerous tissues as compared with matched surrounding tissues, and its protein expression was greater in HCC cells than the non-transformed hepatic cell line L02. Furthermore, upregulation of CSIG significantly increased the colony formation of SMMC7721 and HepG2 cells, and silencing CSIG could induce cell cycle arrest and cell apoptosis. The tumorigenic ability of CSIG was confirmed *in vivo* in a mouse xenograft model. Our results showed that CSIG promoted the proliferation of HepG2 and SMMC7721 cells *in vivo*. Finally, CSIG protein directly interacted with c-MYC protein and increased c-MYC protein levels; the ubiquitination and degradation of c-MYC protein was increased with knockdown of CSIG. CSIG could also increase the expression of c-MYC protein in SMMC7721 cells *in vivo*, and it was noted that the level of c-MYC protein was also elevated in most human cancerous tissues with high level of CSIG.

## INTRODUCTION

Hepatocellular carcinoma (HCC) is the most common type of primary liver cancer and the third-leading cause of cancer deaths worldwide [1]. Extensive studies have shown that the major risk factors of HCC are chronic viral hepatitis, alcoholism, nonalcoholic fatty liver disease, and exposure to environmental agents such as aflatoxins [2]. However, the genetic and biochemical events controlling the development and progression of HCC are still fragmented [3]. Currently, surgical resection or liver transplantation is the treatment for HCC [4], but a large number of patients with advanced disease or older patients are not suitable for surgery [5]. The activation of oncogenes and the loss of tumor suppressor genes are believed to play key roles in the pathogenesis of HCC [5]. Therefore, discovery of new genes related to HCC and understanding their mechanism may provide important clues for HCC treatment.

Cellular senescence-inhibited gene (CSIG), also named ribosomal L1-domain-containing 1 (RSL1D1), belonging to the L1p/L10e family [6], was autonomously cloned from human diploid fibroblast cells 2BS by our laboratory (Genebank accession no. AY154473) [7]. The human CSIG gene is located on chromosome 16p13.3 and is composed of 9 exons [6]. CSIG protein, composed of 490 amino acids, has a ribosomal L1 domain in its N terminus and a lysine-rich domain in its C terminus [6]. CSIG is abundantly expressed in growing human diploid fibroblast cells, but its expression declines during cellular senescence [6, 7]. In addition, the proliferation of Sprague-Dawley rat tenocytes decreases with age, which is associated with downregulated CSIG [8]. The transcription of CSIG is repressed by Rb-mediated heterochromatin via a TAAC DNA element during cellular senescence [9]. CSIG can significantly delay cell senescence by inhibiting phosphatase and tensin homolog protein (PTEN) translation [6]. In addition to interacting with PTEN mRNA, CSIG interacts with other proteins such as p33ING1 and nucleostemin [10, 11]. CSIG protein significantly prolongs the progression of replicative senescence; however, its roles in HCC are not clear.

c-MYC proteins (hereafter referred to as MYC) are basic helix-loop-helix leucine zipper (HLH-ZIP) transcription factors. MYC forms heterodimeric complexes with Max, another HLH-ZIP protein, and binds to 5′-CACGTG-3′ and similar E-box DNA sequences for transcription of target genes [12]. Overexpression of MYC promotes oncogenic transformation and tumorigenesis by activating the transcription of target genes that drive cell proliferation and stimulate angiogenesis and repress cell differentiation [13]. *MYC* is frequently overexpressed in HCC [14, 15, 16]. Tissue microarray analysis showed that up to 70% of human virus and alcohol-related HCC shows excessive activation of MYC [14]. Furthermore, studies of HCC patients with hepatitis B virus infection revealed that MYC could be activated by HBx protein, which enhanced HBx-mediated carcinogenicity [17]. In contrast, downregulation of MYC inhibited the growth of HepG2 and Morris 5123 liver cancer cells [18, 19]. Therefore, studies of CSIG regulation of MYC and its downstream genes could significantly elucidate the relationship between CSIG and HCC proliferation.

This study aimed to investigate in-depth the effects of CSIG on HCC growth and the underlying mechanism to explore the possibility of CSIG suppression in clinical treatment of HCC.

## RESULTS

### Increased expression of CSIG in HCC

**Figure 1:**
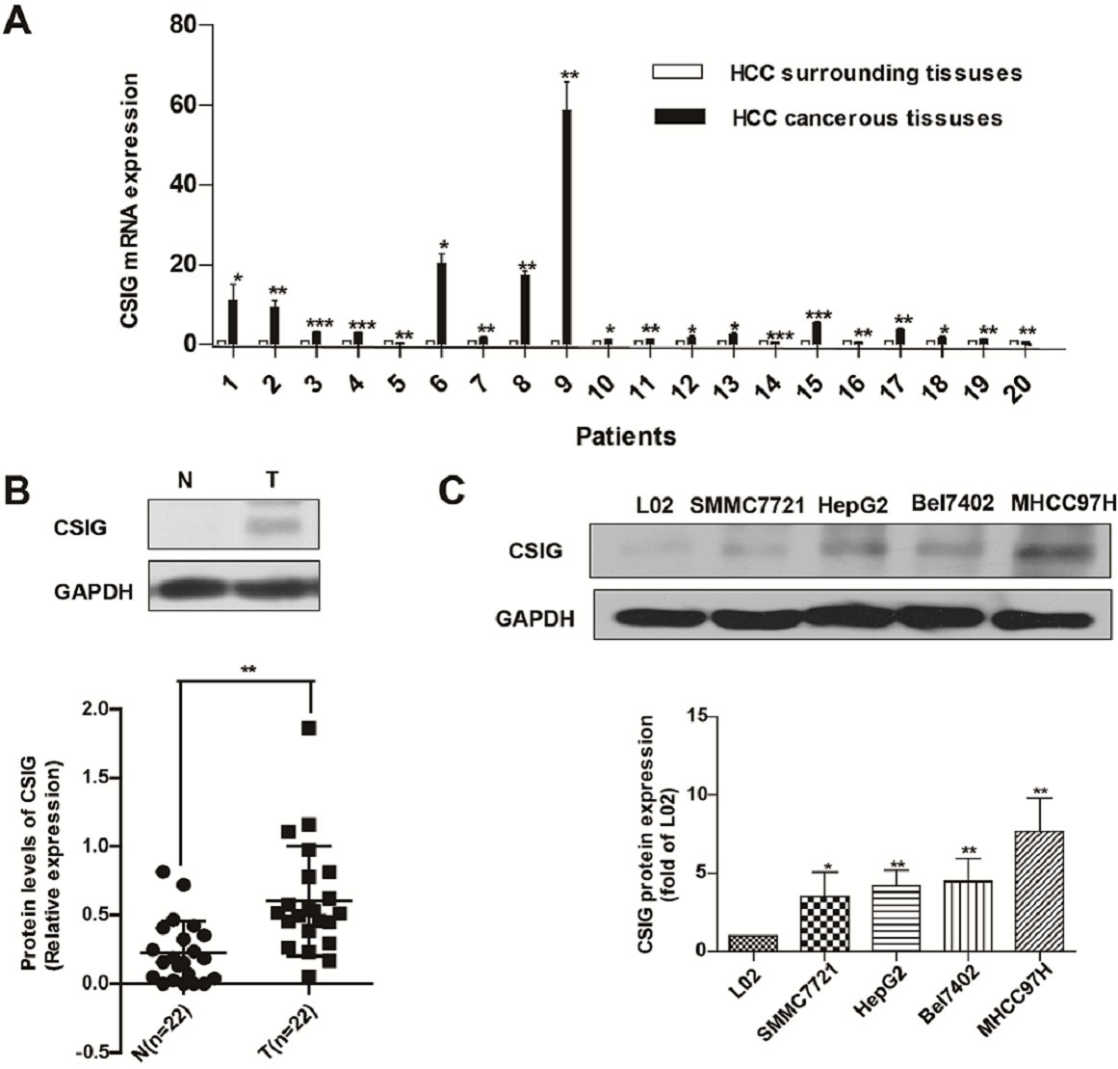
Increased expression of CSIG in HCC is associated with MYC protein **(A)** Quantified real-time PCR analysis of the mRNA expression of CSIG in 20 paired clinical specimens. **(B)** Western blot analysis of CSIG and MYC protein levels in 22 paired clinical HCC samples and surrounding liver tissues and quantification. (N, adjacent non-tumor tissues; T, HCC tissues) **(C)** Western blot analysis of CSIG protein levels in HCC cells and the hepatic cell line L02. *P < 0.05, **P < 0.01 compared with L02 group.

To explore the association of CSIG and HCC, we detected CSIG mRNA in HCC tissues and adjacent non-tumor tissues from 20 patients. CSIG mRNA levels were higher in most HCC tissues than adjacent tissues (Figure 1A).

We also detected protein levels of CSIG in HCC tissues and adjacent non-tumor tissues from 22 patients. CSIG protein levels were significantly higher in HCC tissues than that in adjacent non-tumor tissues (0.23 versus 0.60), (*P* < 0.01, Figure 1B). CSIG protein levels were increased in 86.4% (19/22) of HCC samples compared with adjacent non-tumor tissues and decreased in 13.6% (3/22) of HCC samples (Tables 1, 2). However, whether the expression of α-fetoprotein (AFP) was positive or negative in patients' serum, CSIG protein levels were higher in most HCC samples than in adjacent non-tumor tissues (Tables 1, 2). Then we used Spearman method to analyze the correlation between AFP serum levels and CSIG expression in HCC tissues. We found that CSIG protein levels in HCC specimens was not associated with levels of serum AFP (*r* = 0.011, *P* = 0.481 > 0.05; Figure S1).

**Table 1:** List of 22 pairs HCC specimens

Patient	Age	Sex, F/M	α-fetoprotein level, μg/L	Tumor size (cm^3^)	Cancer embolus	Liver fibrosis, cirrhosis or necrosis
1	65	F	113.3	6 × 5 × 4	No	necrosis
2	63	M	19463	12.0 × 9.0 × 6.5	Yes	fibrosis and necrosis
3	68	M	2.25	7.5 × 6.0 × 5.5	No	No
4	40	F	9621	12 × 9 × 5	Yes	No
5	74	M	4.21	3.5 × 2.6 × 1.5	No	No
6	56	F	292.7	2.7 × 2.5 × 2.5	No	No
7	62	M	3.23	3.5 × 3.3 × 4.5	No	No
8	36	M	6.36	3.5 × 3 × 3	Yes	No
9	62	M	4.68	3.5 × 0.6 × 0.6	No	cirrhosis
10	60	M	1000	12 × 10 × 8	No	cirrhosis
11	57	M	1000	15	Yes	cirrhosis
12	75	M	1.4	8 × 6	No	cirrhosis
13	43	M	1189	6 × 5 × 5	Yes	cirrhosis
14	75	M	75	3	No	cirrhosis
15	60	F	1024	2.5 × 2.5 × 2.0	No	cirrhosis
16	78	M	2.74	11 × 10 × 8	No	cirrhosis
17	74	M	2.63	5 × 5 × 3	No	cirrhosis
18	74	M	1.3	7 × 6 × 6	No	cirrhosis
19	69	F	1210	6 × 6 × 5	No	cirrhosis
20	39	M	1210	20 × 20 × 15	Yes	cirrhosis
21	60	F	1000	4.5 × 4.5 × 4.0	No	cirrhosis
22	73	F	6.95	No data	Yes	No data

**Table 2:** Protein levels of CSIG and serum AFP in clinical specimens

CSIG protein (T/N)	α-fetoprotein level, μg/L		Total
	Low (<300)	High (≥1000)	
Overexpression	10	9	19
No overexpression	3	0	3
Total	13	9	22

N, adjacent non-tumor tissues; T, HCC tissues

Furthermore, we measured CSIG protein expression in 4 liver cancer cell lines (SMMC7721, HepG2, Bel7402 and MHCC97H); the control was normal L02 liver cells, an immortalized human liver cell line. The relative expression of CSIG was 3.53-, 4.23-, 4.48-, and 7.69-fold, respectively, in the 4 HCC cells as compared with that of L02 cells (Figure 1C). The metastatic and aggressive ability of MHCC97H is high, but metastatic and aggressive abilities of SMMC7721, HepG2, Bel7402 cells are very low. We found that the expression of CSIG in 97H cells was much higher than other three HCC cells (SMMC7721, HepG2 and Bel7402). So CSIG might correlate with metastatic potential of HCC cells.

Thus, the upregulation of CSIG is a frequent event in HCC and tumor cell lines.

### Effect of CSIG on colony formation of HCC cells *in vitro*

**Figure 2:**
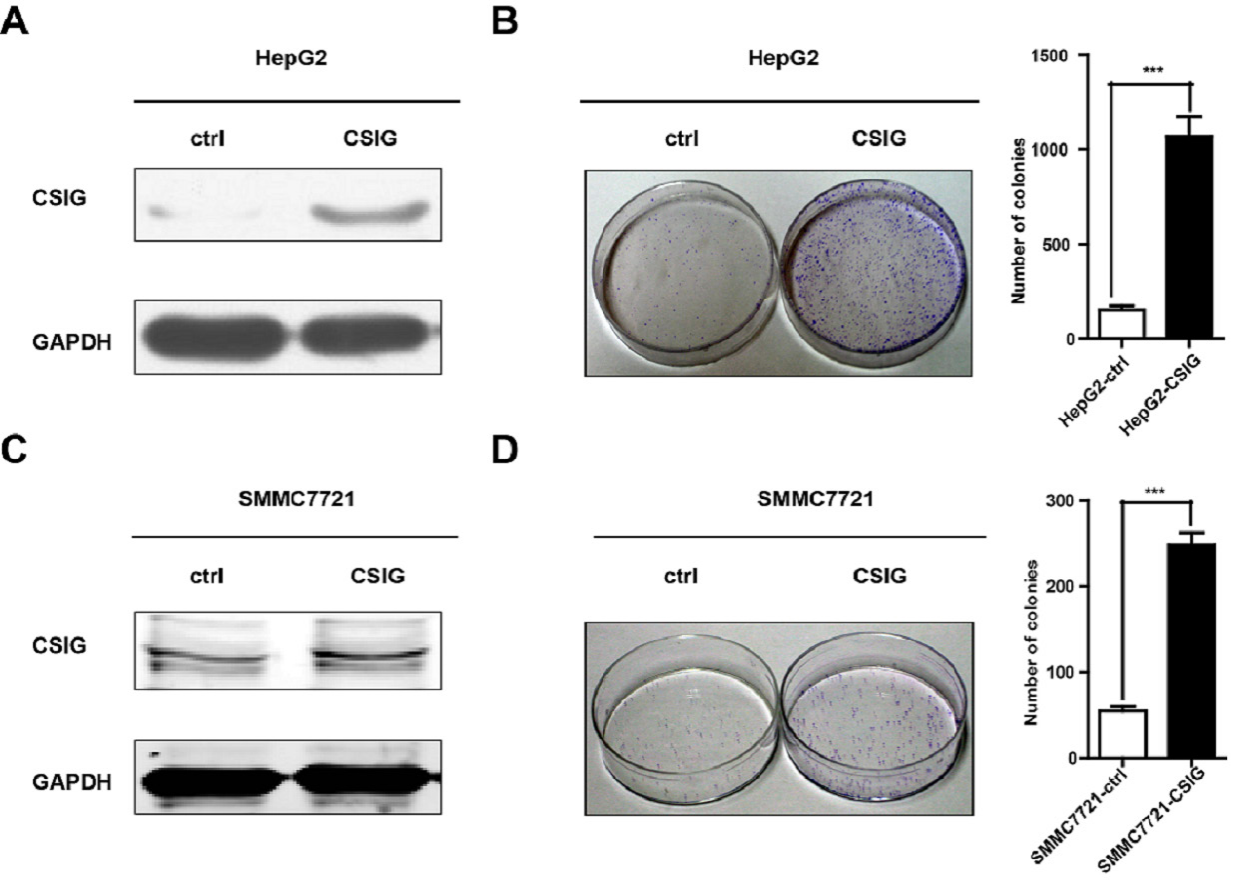
CSIG promoted the colony formation of HCC cells *in vitro* **(A)** Western blotting analysis of stable transfection of CSIG or control vector in HepG2 cells. **(B)** Colony formation assays of HepG2 cells with upregulated CSIG. **(C)** Western blotting analysis of stable transfection of CSIG or control vector in SMMC7721 cells. **(D)** Colony formation assays of SMMC7721 cells with upregulated CSIG. ***P < 0.001 compared with ctrl group.

To investigate the biological function of CSIG in HCC proliferation, we overexpressed CSIG in tumor cells by retrovirus mediation, and then established 2 stable CSIG-overexpression cell lines, HepG2-CSIG and SMMC7721-CSIG. CSIG protein was significantly upregulated in HepG2-CSIG and SMMC7721-CSIG cells as compared with control cells (Figure 2A, Figure 2C). Upregulation of CSIG significantly increased the colony formation of SMMC7721 and HepG2 cells (*P* < 0.001, Figure 2B and Figure 2D). Therefore, CSIG functions as a tumor promoter and controls the growth of HCC cells *in vitro*.

### CSIG silencing causes cell cycle retardation and apoptosis of HCC cells

**Figure 3:**
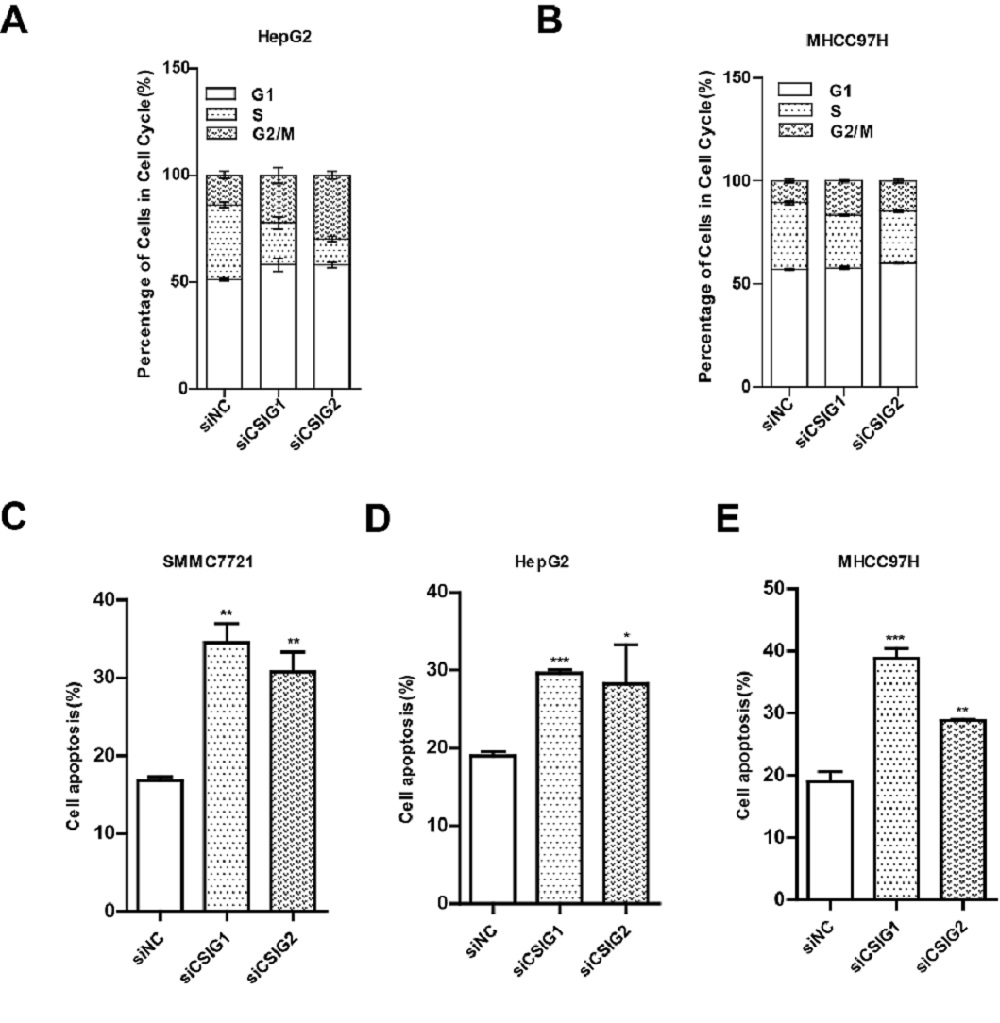
CSIG-silencing induced cycle arrest and cell apoptosis **(A)** Flow cytometry of cell cycle of HepG2 cells transfected with small interfering negative control (siNC), siCSIG1 or siCSIG2 for 72 h. **(B)** MHCC97H cells. **(C)** Apoptotic analysis of SMMC7721 cells treated with small interfering negative control (siNC), siCSIG1 or siCSIG2 for 72 h. **(D)** HepG2 cells. **(E)** MHCC97H cells. **(C,D,E)** Data were expressed as mean ± SD. *P < 0.05, **P < 0.01, ***P < 0.001 compared with siNC group.

To clarify how CSIG promotes cell growth, we analyzed the cell cycle of HepG2 and MHCC97H cells transfected with CSIG siRNA1 (siCSIG1) and CSIG siRNA2 (siCSIG2) for 72 h. HepG2 cells accumulated at the G1 phase (from 51.4% to 58.2% or 58.1%) and G2/M phase (from 13.9% to 22.3% or 29.7%) with a concomitant depletion of S-phase cells (from 34.9% to 19.6% or 12.2%) with siCSIG transfection (Figure 3A). Meanwhile, MHCC97H cells accumulated at the G2/M phase with a concomitant depletion of S-phase cells with siRNA transfection, with no change in G1 phase of MHCC97H cells (Figure 3B). The apoptosis rate of 3 HCC cells (SMMC7721, HepG2, and MHCC97H) significantly increased with siCSIG transfection (Figure 3D–3E). Therefore, CSIG silencing induced depletion of cells in the S phase and apoptosis in HCC cells.

### CSIG affects MYC protein expression

**Figure 4:**
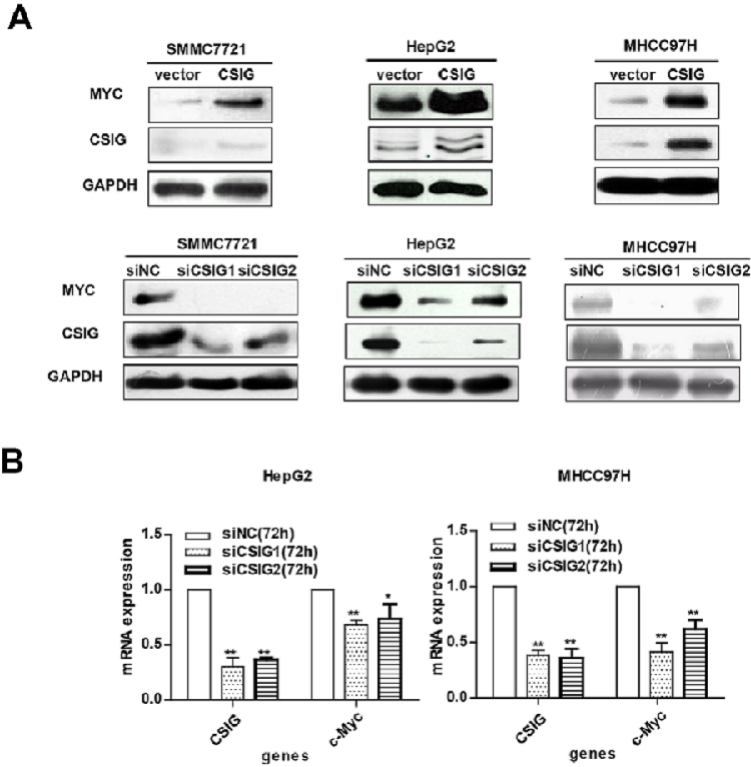
CSIG promoted the expression of MYC protein **(A)** Western blot analysis of CSIG and MYC protein expression with CSIG overexpression and siCSIG1 and siCSIG2 knockdown in HCC cells. **(B)** Quantified MYC mRNA expression.

To clarify how CSIG affects the proliferation and cell cycle of HCC cells, we examined the protein levels of a number of important tumor-related genes, including MYC, PTEN, and so on. CSIG affected the protein expression of MYC in 3 HCC cells (Figure 4A) but not PTEN (Figure S2A–S2B). MYC mRNA levels were decreased ~0.32 and ~0.58 times in HepG2 and MHCC97H cells, respectively, with siCSIG1 transfection (Figure 4B). However, MYC protein was almost not detected in MHCC97H cells and was very low in HepG2 cells with siCSIG1 transfection (Figure 4A). Thus, CSIG regulation of MYC mainly depends on protein levels.

### CSIG interacts with MYC *in vitro* and *in vivo*, and knockdown of CSIG promotes MYC protein degradation

**Figure 5:**
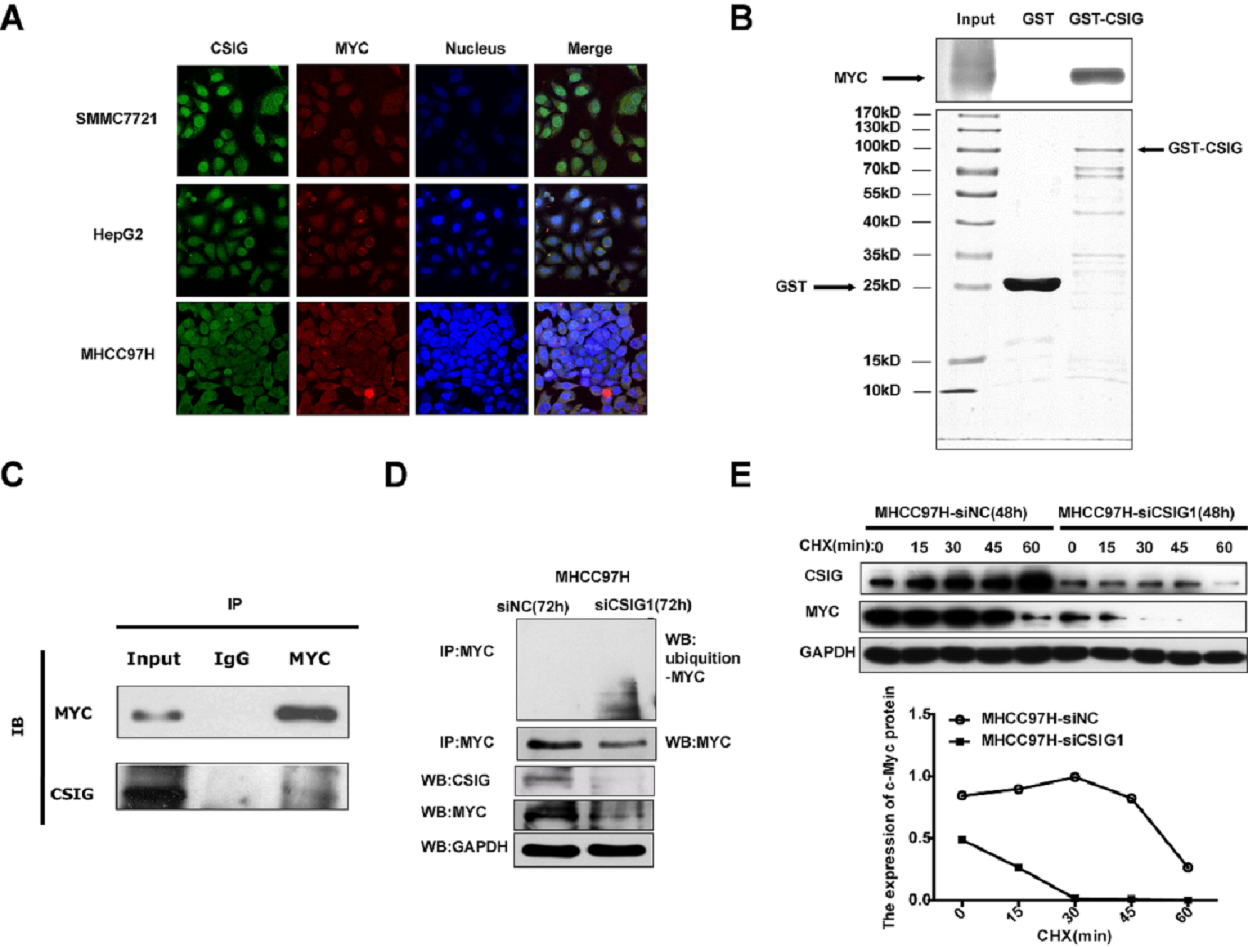
CSIG interacted with MYC *in vitro* and *in vivo*, and knockdown of CSIG promoted MYC protein degradation **(A)** The location of CSIG and MYC protein in 3 HCC cells. **(B)** GST-pull down assay of the interaction of CSIG protein and MYC protein *in vitro*. **(C)** Immunoprecipitation of CSIG and MYC protein in SMMC7721 cells using the MYC antibody. **(D)** CSIG knockdown promoted ubiquitination of MYC protein in MHCC97H cells. **(E)** CSIG knockdown promoted degradation of MYC protein in MHCC97H cells.

Because CSIG locates and interacts with nucleostemin and p33ING1 [10, 11], it might interact with MYC protein. Immunofluorescence analysis confirmed that both CSIG and MYC protein were mainly located in the nucleus and was also detected in the cytoplasm in 3 HCC cell lines SMMC7721, HepG2 and MHCC97H (Figure 5A). Next, we tested whether CSIG and MYC interacted with each other *in vitro* by GST pull down experiments. We performed a GST pull down assay using *in vitro* purified GST-CSIG protein and translated MYC protein. This assay showed that CSIG displays a strong association with MYC (Figure 5B). Hence, the direct interaction between CSIG and MYC was demonstrated. Next, we tested whether CSIG interacted with MYC *in vivo* by immunoprecipitation experiments. Endogenous CSIG protein was immunoprecipitated by MYC in SMMC7721 cells (Figure 5C).

Many studies have reported that the regulation of MYC protein turnover via the ubiquitin-proteasome pathway is the major mechanism controlling functions of MYC [20], so we detected the impact of silencing CSIG on ubiquitination of MYC. The relative expression of CSIG was higher in MHCC97H than SMMC7721 and HepG2 cells (Figure 1C), so we analyzed the effect of silencing CSIG on MYC ubiquitination in MHCC97H cells. Compared to MHCC97H-siNC cells, MHCC97H-siCSIG1 cells showed enhanced ubiquitination (Figure 5D). Since MYC protein turnover is quick, we examined whether CSIG knockdown affected MYC protein turnover. We treated cells with 50 μM cycloheximide (CHX), an inhibitor of new protein synthesis, at different times after transfection with negative control siRNA (siNC) or siCSIG1 for 1 h. The MYC protein half-life was reduced from ~50 min in MHCC97H cells transfected with siNC to ~15 min in cells transfected with siCSIG1 (Figure 5E). Therefore, knockdown of CSIG increased the ubiquitin-proteasome pathway-mediated MYC degradation and led to downregulation of MYC protein.

We suggest the following regulatory pathway: knockdown of CSIG in HCC cells promotes ubiquitination of MYC, which leads to MYC degradation. Downregulation of MYC inhibits cell cycle and growth.

### CSIG affects tumor growth in a HCC xenograft transplantation model

**Figure 6:**
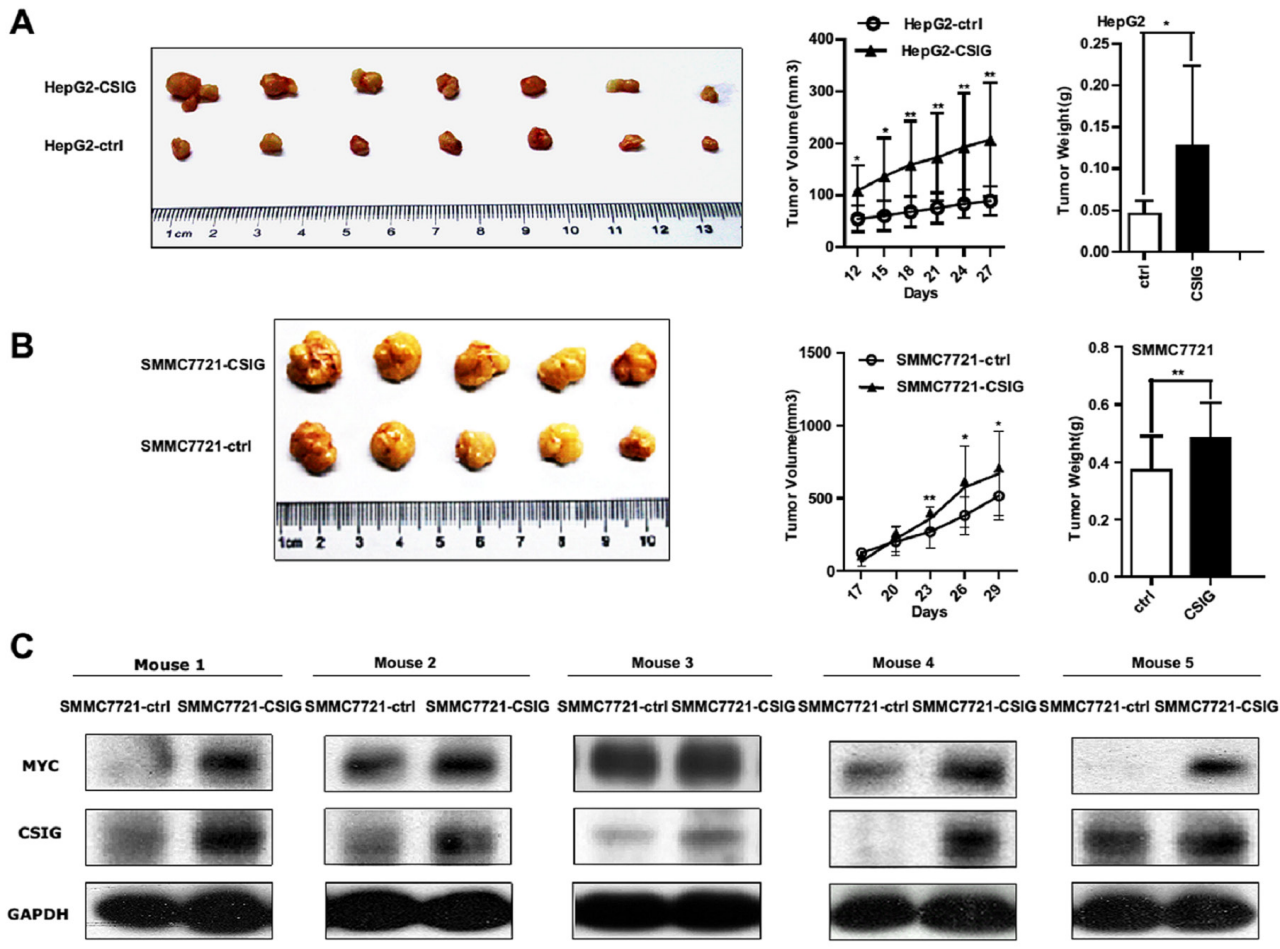
CSIG affected tumor growth of HCC cells in nude mice and MYC protein level *in vivo* **(A)** The tumor growth was increased with upregulated CSIG in HepG2 cells. **(B)** Tumor growth of SMMC7721 cells with upregulated CSIG in nude mice. **(A,B)** Values were mean±SD. *P < 0.05, **P < 0.01 compared with control group.**(C)** Expression of CSIG and MYC in tumor tissues treated with SMMC7721-CSIG and SMMC7721-ctrl cells.

Because our *in vitro* studies suggested a functional role for CSIG in HCC proliferation and cell cycle, we investigated the contribution of CSIG to HCC growth *in vivo*. HepG2 and SMMC7721 cells transduced with empty vector (pBabe) or CSIG-expressing lentivirus were subcutaneously injected into Balb/c nude mice, and tumor growth was monitored. The mean tumor volume and weight was increased in the CSIG-infected group (Figure 6A–6B). At 27 or 29 days after inoculation, mice injected with HepG2-CSIG and SMMC7721-CSIG cells carried a larger burden (Figure 6A–6B).

### Activation of MYC by CSIG *in vivo*

Because our *in vitro* studies suggested that CSIG could activate MYC protein expression, we detected whether CSIG promoted MYC protein level *in vivo*. The protein level of CSIG and MYC was greater in tumor tissues of mice treated with SMMC7721-CSIG than SMMC7721-control (Figure 6C).

**Figure 7:**
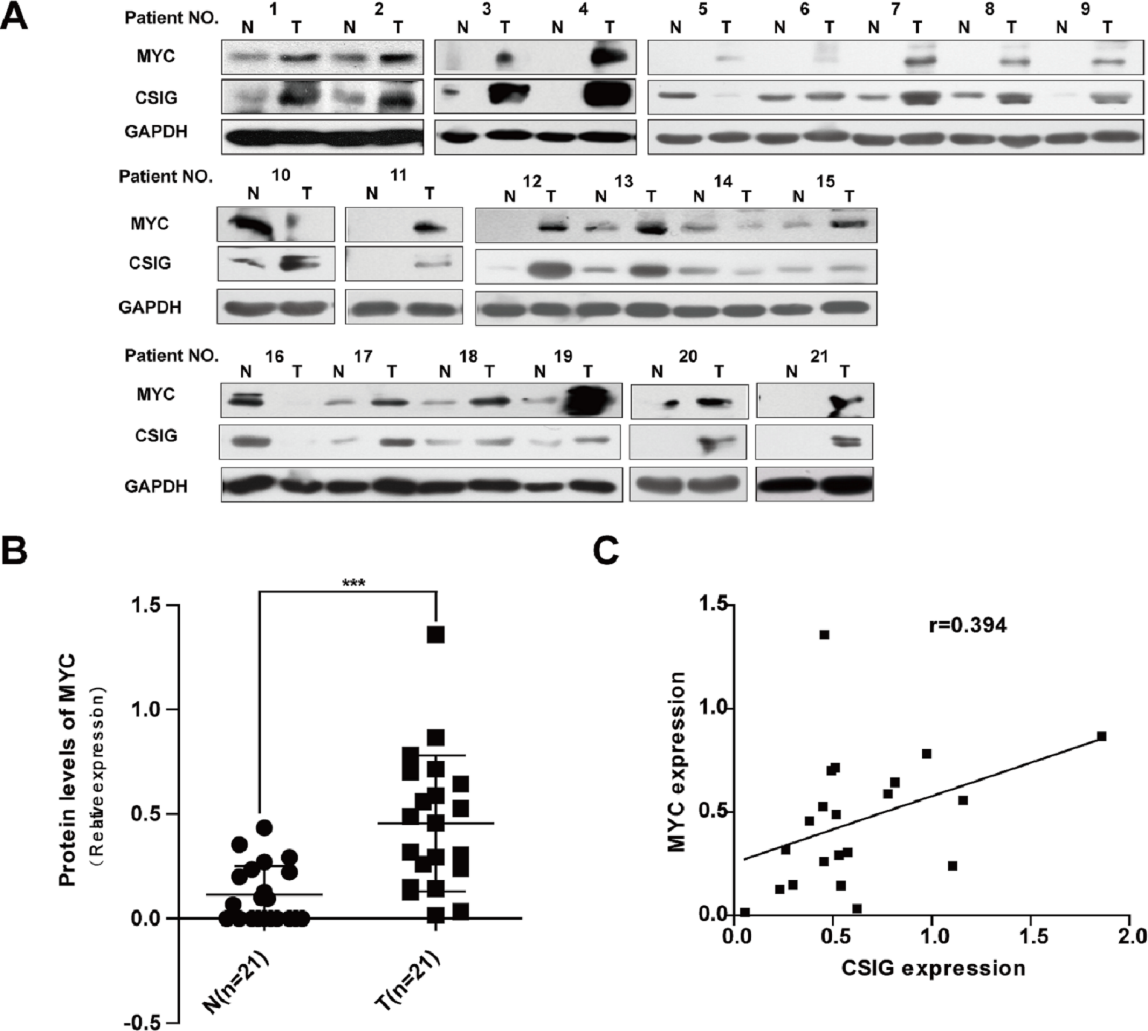
Increased expression of CSIG in HCC was associated with MYC protein **(A)** Western blot analysis of CSIG and MYC protein levels in ~21 paired clinical HCC samples and surrounding liver tissues. **(B)** Quantification of MYC protein levels in 21 paired clinical HCC samples and surrounding liver tissues. (N, adjacent non-tumor tissues; T, HCC tissues). ***P < 0.001 compared with N group. **(C)** Statistically significant association between CSIG and MYC expression at protein levels in HCC specimens (*r* = 0.394, *P* < 0.05).

Meanwhile, we also examined the expression of oncoprotein MYC in HCC specimens. MYC protein levels were increased in 85.7% (18/21) of HCC samples compared with adjacent non-tumor tissues (Figure 7A; Table 3). MYC protein levels were significantly higher in HCC tissues than that in adjacent non-tumor tissues, (0.11 versus 0.46), (*P* < 0.01, Figure 7B). It was noted that the level of MYC protein was also elevated in most human cancerous tissues with high level of CSIG (Figure 7A; Table 3). Then, we assessed whether CSIG would be implicated in MYC expression. A significant positive correlation between the protein expression of CSIG, and that of MYC, was observed in the same 21 HCC tissues. (*r* = 0.394, *P* < 0.05; Figure 7C).

**Table 3:** Association of cases of CSIG and MYC expression

CSIG protein (T/N)	MYC protein (T/N)		Total
	Overexpression	No overexpression	
Overexpression	17	1	18
No overexpression	1	2	3
Total	18	3	21

N, adjacent non-tumor tissues; T, HCC tissues

These results provide strong evidence that CSIG may promote tumorigenesis of hepatoma cells *in vivo* by regulating MYC protein expression.

## DISCUSSION

Hepatocarcinogenesis is a multistep process that involves multiple factors including oncogenes [15]. CSIG was autonomously cloned from human diploid fibroblast cells by our own laboratory (Genebank accession no. AY154473), however its role in tumorigenesis is unknown [7]. In this study, we first report the role of CSIG in tumor growth and the relationship between MYC and CSIG.

Firstly, we reported a stronger expression of CSIG protein in HCC tissues than adjacent non-tumor tissues and in HCC cell lines than the nontransformed hepatic cell line L02 (Figure 1). CSIG protein levels were increased in 86.4% of HCC tissues compared with adjacent tissues. CSIG protein was very low or was almost not detected in most adjacent non-tumor tissues. CSIG may be an attractive therapeutic target for HCC because of its upregulation in liver tumor and low expression in adjacent non-tumor tissues.

AFP has been a useful biomarker for diagnosis of HCC since the 1970s and currently is widely used in the clinical diagnosis of liver cancer [21]. Recently, the diagnostic value of serum AFP is questioned recently because of its low sensitivity [22]. Singal et al. found a large gap between the efficacy and effectiveness of AFP for HCC surveillance among patients with cirrhosis [22]. Interestingly, CSIG protein expression in HCC specimens was not related to levels of serum AFP (Figure S1), and among 13 of our HCC patients with low levels of serum AFP, for 10, CSIG was overexpressed in HCC tissues (Tables 1, 2).

Considering the high expression of CSIG in HCC tissues and tumor cells, we examined whether CSIG could facilitate the tumorigenesis of HCC. Cell proliferation and colony number were increased in SMMC7721 and HepG2 cells with CSIG overexpression (Figure 2A–2D). In addition, knockdown of CSIG in HCC cells induced significant cell depletion in the S phase and apoptosis, which suggested that reduced CSIG level perturbed the cell cycle progression and cell apoptosis of HCC cells, thus inhibiting their proliferation (Figure 3A–3E).

To clarify how CSIG affects the proliferation and cell cycle of HCC cells, we examined the protein levels of a number of important tumor-related genes, including MYC and PTEN. CSIG could promote MYC protein expression in 3 HCC cells (Figure 4A) but did not affect PTEN (Figure S2) levels. CSIG not affecting PTEN disagrees with Ma et al. [6], who found that CSIG could decrease PTEN protein level by inhibiting its translation in 2BS and HEK293 cells. This discrepancy could be explained by different cell types.

Further we examined how CSIG affected MYC protein expression. CSIG protein co-exists and interacts with nucleostemin and p33ING1 protein [10, 11], therefore, we performed immunofluorescence, GST-pull down and immunoprecipitation analysis to detect whether CSIG interacted with MYC. Some previous studies showed that CSIG located in nucleolus in HEK293 cells [6, 11]. We found that CSIG mainly located in the nucleus and was also detected in the cytoplasm in all HCC cells (Figure 5A). CSIG directly interacted with MYC *in vivo* and endogenous CSIG protein was immunoprecipitated by MYC in SMMC7721 cells (Figure 5B–5C). Nucleolar proteins shuttling between the nucleolus and nucleoplasm are important for many non-ribosomal processes including regulation of cell growth and death, stress responses and the cell cycle [23, 24, 25]. Thus CSIG involved in regulating HCC cell growth and cell cycle might be associated with its movement from the nucleolus to the nucleoplasm. CSIG mainly locates in the nucleus in all HCC cells, so CSIG could interact with MYC, which is also mainly located in the nucleus in the 3 HCC cells, SMMC7721, HepG2 and MHCC97H (Figure 5A).

Because the regulation of MYC protein turnover via the ubiquitin-proteasome pathway is the major mechanism controlling functions of MYC [20], we detected the effect of silencing CSIG on the ubiquitination and half-life of MYC in HCC cells. With CSIG silenced, the ubiquitination of MYC was greatly elevated, whereas the half-life of MYC was greatly decreased (Figure 5D–5E). F-box protein Fbw7 and ubiquitin ligase skp2 regulate the stability of MYC proteins by interacting with MYC and promoting its ubiquitination [26, 27]. So we supposed that the interaction of CSIG and MYC might interfere with the interaction of MYC and Fbw7 or skp2, then change the ubiquitination and stability of MYC protein. There are several possible regulatory mechanisms of CSIG regulating MYC protein and further studies will be required to elucidate the possibilities.

MYC is among the most potent oncogenes associated with HCC development in mice [28]. Therefore, we performed subcutaneous xenograft experiments to discern the molecular mechanism involved in activated MYC by CSIG *in vivo*. CSIG promoted tumor growth of HepG2 cells in nude mice (Figure 6A). Importantly, CSIG-infected SMMC7721 cells formed larger tumors and showed higher expression of MYC as compared with controls, which corresponded with *in vitro* experiments (Figure 6B–6C). We also examined the expression of MYC and CSIG at protein levels in clinical specimens from 21 patients. MYC expression was higher in 19 of the 21 HCC tissues (85.7%) than adjacent non-tumor tissues (Figure 7A; Table 3). The frequency of MYC overexpression in our experiments was higher than that observed in a study by Li et al. (85.7% versus 74.2%) (Figure 7A; Table 3) [15]. This discrepancy could be attributed to clinical specimens or methodical differences between the studies. It was noted that the level of c-MYC protein was also elevated in many human cancerous tissues with high level of CSIG (Figure 7A; Table 3). A significant positive correlation between the protein expression of CSIG, and that of MYC, was observed in the same 21 HCC tissues. (Figure 7C). These results provide strong evidence to support that CSIG promoted tumorigenesis of hepatoma cells *in vivo* by increasing MYC protein levels in liver.

In summary, this study demonstrated a previously unknown role of CSIG having a promotion effect in cell proliferation in HCC. The over-expression of CSIG in HCC was positively correlated with MYC protein. CSIG enhanced the expression of the oncoprotein MYC; downregulation of CSIG boosted the ubiquitination of MYC and thus accelerated its degradation. Our results provide new insights into the pathogenesis of HCC. Interruption of the CSIG-MYC pathway may be a promising strategy for HCC.

## MATERIALS AND METHODS

### Clinical specimens

Human HCC tissues and corresponding surrounding liver samples from 22 Chinese patients were obtained from the Tissue Bank at the Peking University School of Oncology (patients 1–9) and Henan Cancer Hospital (patients 10–22). All samples, collected from surgical resection, were snap-frozen in liquid nitrogen and stored at −80°C. The clinical pathologic characteristics of the patients are summarized in Table 1. All human samples were collected in accordance with the Declaration of Helsinki, and use of human tissues was approved by the Institute Research Ethics Committee at the 2 hospitals. Informed consent was obtained from all patients.

### Cell culture

Human HCC cell lines HepG2, MHCC97L, MHCC97H [29], and Bel7402 and the packaging cell line Phoenix were cultured in high-glucose DMEM supplemented with 10% fetal bovine serum (FBS) at 37°C in 5% CO_2_. SMMC7721 and L02 cells were cultured in RPMI 1640 media containing 10% FBS at 37°c in 5% CO_2_ [30]. The source of cells is in Supplementary materials and methods.

### Transient transfection

For gene over-expression experiments, plasmid transfections were performed by using Lipofectamine 2000 reagents (Invitrogen, Carlsbad, USA) for cells, and analyzed 48 h or 72 h after transfection. For siRNA knockdown assays, siRNA targeting CSIG were 5′-AGAAGGAACAGACCCCAGA-3′ and 5′-AGUG GUUCUUGCAGUGCUA-3′, and negative control siRNA was 5′-UUCUCCGAACGUGUCACGU-3′ (Table S1). Cells were transfected with siRNA duplexes for 48 h or 72 h by using the Lipofectamine RNAiMAX (Invitrogen, Carlsbad, USA).

### Quantitative-PCR

According to the manufacturer's protocols, total tissue RNA were extracted, and first strand cDNA was synthesized using the Transgen first strand cDNA synthesis kit (Transgen Biotec Co. Ltd., Beijing, China). Quantitatative-PCR was performed to amplify CSIG, MYC, 18S ribosomal RNA (18S rRNA) and glyceraldehyde-3-phosphate dehydrogenase (GAPDH) by using the primers listed in Table S2. Quantitative-PCR was performed in triplicate by using the SYBR Green PCR master mix (Applied Biosystems, ABI) on an ABI 7500 Real Time PCR System. The 18S rRNA or GAPDH was served as an endogenous control for normalization, and fold changes were calculated by means of relative quantification (2^−ΔΔct^).

### GST-pull down assays

Escherichia coli strain BL21 was used to produce Glutathione S transferase (GST) or GST-CSIG fusion proteins. *In vitro*-translated c-MYC protein was generated using the TNT-coupled Transcription/Translation system (Promega, USA). MYC protein were incubated with GST or GST-CSIG bacterial recombinant protein immobilized on glutathione-conjugated Sepharose 4B bead at 4°C for 2 h. Beads were then washed five times in 1 ml wash buffer (428ml H2O, 30ml 2.5M Nacl, 25ml 1M Tris-Hcl PH7.5, 15ml 10% NP-40, 2ml 0.5M EDTA). The bound proteins were eluted with 2 × SDS sample buffer, fractionated by SDS-PAGE and subjected to western blot analysis with anti-MYC antibody.

### Immunoprecipitation assays

SMMC7721 cells were collected from two plates 10 cm in diameter by use of IP lysate buffer which contained protease inhibitor cocktail. Lysates were incubated with monoclonal anti-MYC (5605S, Cell Signaling) overnight at 4°C, then protein-G sepharose beads (Millipore) for another 2 h at 4°C. Immunoprecipitates were washed 4 times with immunoprecipitation lysate buffer and resuspended in 90 μl 2 × SDS loading buffer. The samples were boiled for 10 min and determined by CSIG and MYC antibody.

### *In vivo* ubiquitination assays

MHCC97H cells were transfected with negative control siRNA (siNC) or siCSIG1. 72 h after transfection, cellular proteins were extracted, total cell lysates were then immunoprecipitated with anti-MYC antibody and protein-G sepharose beads (Millipore). Eluted proteins were immunoblotted with anti-ubiquitination antibody.

### MYC protein half-life assays

MHCC97H cells were transfected with siNC or siCSIG1 for 48 h, and treated with 50μM cyclohexamide (CHX) for the last 0, 15, 30, 45 and 60 min in MHCC97H-siNC and MHCC97H-siCSIG1 cells. Proteins were extracted from the cells and subjected to western blotting analysis of the MYC protein. MYC protein levels were normalized by GAPDH, and half-life (T_1/2_) of MYC proteins were obtained from the line chart.

### Tumor xenografts

HCC cells (about 3 × 10^6^) were resuspended in 200 μL phosphate buffered saline (PBS) and injected subcutaneously into the left or right side of each BALB/c nude mice. One side was implanted with control tumor cells, and the other was implanted with tested tumor cells. Tumor sizes were measured every few days, and tumor volumes were calculated as volume = length × width^2^ × (1/2). After several weeks, mice were killed and tumors were harvested. All procedures were approved by the Institutional Animal Care and Use Committee.

### Statistical analysis

Data were shown as mean ± SD from at least 3 independently performed experiments. Student's *t* test was used for analysis. *P* < 0.05 was considered statistically significant. Correlation analysis involved the Spearman method.

### Other materials and methods

For a description of other materials and methods in this study, see the Supplementary Information.

## SUPPORTING MATERIALS AND METHODS



## Abbreviations

HCC: hepatocellular carcinoma
CSIG: cellular senescence-inhibited gene
FBS: fetal bovine serum
GAPDH: glyceraldehyde-3-phosphate-dehydrogenase
AFP: α-fetoprotein
18S rRNA: 18S ribosomal RNA
PTEN: phosphatase and tensin homolog
PBS: phosphate buffered saline
GST: Glutathione S transferase
