# Supplementary material for: CSIG promotes hepatocellular carcinoma proliferation by activating c-MYC expression
Source: Oncotarget. 2015 Feb 28;6(7):4733–44. doi: 10.18632/oncotarget.2900 (PMC4467111; doi:10.18632/oncotarget.2900)
Supplement: Supplementary file 1 [file oncotarget-06-4733-s001.pdf]

SUPPORTING MATERIALS AND METHODS

Cell sources

Human liver cancer cell lines HepG2 were obtained from American Type Culture Collection (ATCC, Manassas, VA), and Bel7402 cells were gifts of the Department of Biology, Peking University Health Science Center (Beijing, China). Liver cancer cell lines MHCC97L and MHCC97H were obtained from the Liver Cancer Institute of Fudan University (Shanghai, China), and SMMC7721 was a gift from Department of Experimental Hematology, Beijing Institute of Radiation Medicine (Beijing, China). The human immortalized hepatocyte cell line L02 was purchased from China Center for Type Culture Collection (CCTCC, Wu Han, China). Phoenix packaging cells were gifts from Dr. M. Narita (Cancer Research UK, Cambridge Research Institute, England).

Stable transfection of CSIG in HCC cells

Plasmids pBabe-CSIG were used for overexpression of CSIG in HCC cells. For stable transfection, retroviral infection was used. After Phoenix cells reached 60% confluence, the retroviral plasmids were transfected with CaCl2 reagent (M&C Gene Technology Ltd., Beijing, China) according to the manufacturer's instructions. The retrovirus supernatants were collected 48 h after transfection and then filtered with o.45 μm filters. HepG2 and SMMC7721 cells were infected with retrovirus in the presence of 8 μg/ml polybrene (Invitrogen, Carlsbad, USA). Pools of stable transformants were obtained by sustained selection for 5 days with 1 μg/ml puromycin (Invitrogen, Carlsbad, USA) for HepG2 cells or 0.8 μg/ml puromycin for SMMC7721 cells.

Western blotting

Proteins were extracted and separated by sodium dodecyl sulfate-polyacrylamide gel electrophoresis (SDS-PAGE). After electric transfer, nitrocellulose filter membrane was sealed with 5% nonfat milk and incubated at 4°C overnight with primary antibodies. After being washed, it was incubated at room temperature for 1 h with second-antibody (1:1000) and colored by ECL.

Blots for celluar total proteins were probed with the following antibodies: anti-CSIG (used as previously described, 1:5000) or (orb10466, biorbyt), anti-c- MYC (5605S, Cell Signaling), anti-PTEN (sc-7974, Santa Cruz Biotechnology), anti-GAPDH (5632–1, Epitomics). Blots for tissue total proteins were probed with anti-CSIG (orb10466, biorbyt) and anti- c-MYC (5605S, Cell Signaling) antibodies.

Colony formation assays

For colony formation assay, 500 or 1000 cells were cultured in 60-mm dishes. Then cells were cultured in DMEM or RPMI 1640 media containing 10% FBS at 37°C in 5% CO2 for 8–10 days. The cells were fixed in 100% methanol for 30 min and were stained with 0.1% Crystal Violet or Giemsa. Colonies with hyper-50 cells were counted, each group had at least triplicate parallel wells.

Flow cytometry for cell cycle and apoptosis assays

For cell cycle assays, cells were washed with PBS, detached with 0.25% trypsin, and fixed with 70% ethanol overnight. After treatment with RNase A (EN0531, Thermo) at 37°C for 30 min, cells were resuspended in PBS and stained with propidium iodide for 10 min. For cell apoptosis assays, cells were collected and then resuspended in binding buffer and stained with Annexin V and propidium iodide in the dark for 30 min. Fluorescence was measured with a FACScan flow cytometry system (BD Biosciences).

Intracellular localization of CSIG and MYC

SMMC7721, HepG2, and MHCC97H cells were seeded in 24-well plates, after 24 h, cells were fixed in 4% paraformaldehyde solution. Rabbit anti-CSIG antibody (orb10466, biorbyt, 1: 1000) and mouse anti-MYC antibody (ab32, Abcam, 1:500) were added and incubated at 4°C. Secondary goat anti-mouse or anti-rabbit IgG antibodies conjugated with rhodamine (TRITC) or fluorescence isothiocyanate (FITC) were applied for 1 h followed by addition of DAPI. Cells were viewed and captured with a Laser Confocal Microscope (Leica TCS-NT SP2, Germany).

SUPPLEMENTARY FIGURES AND TABLES

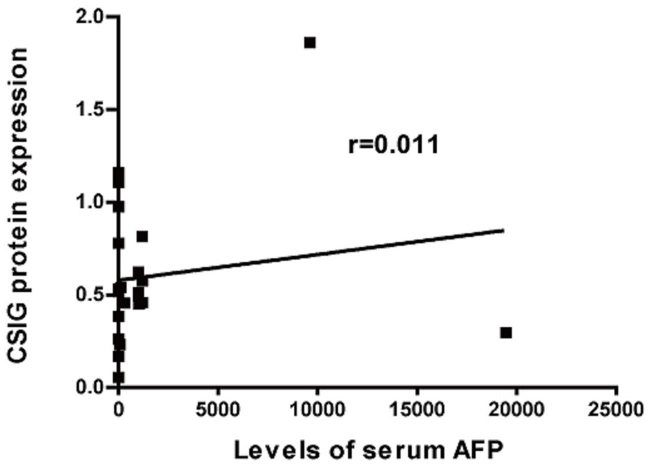

Supplementary Figure S1: Analysis of association between serum AFP levels and CSIG expression in HCC specimens (*r* = 0.011, *P* = 0.481 > 0.05).

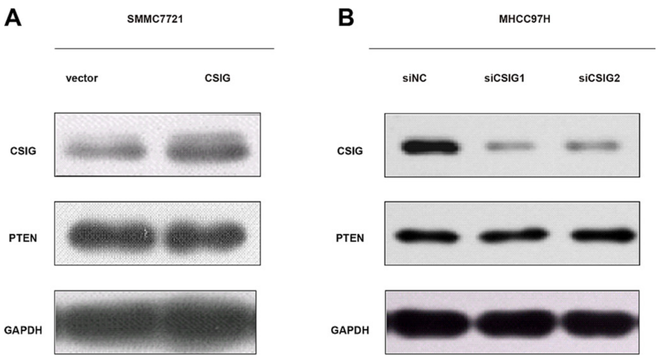

Supplementary Figure S2: CSIG could not affect protein levels of PTEN. (A) Western analysis of PTEN protein levels in SMMC7721-vector and SMMC7721-CSIG cells. **(B)** Western analysis of PTEN protein levels in MHCC97H-siNC, MHCC97H-siCSIG1 and MHCC97H-siCSIG2 cells.

Supplementary Table S1: siRNA target sequences

Name	siRNA target sequences
**siCSIG1**	5′-AGAAGGAACAGACCCCAGA-3′
**siCSIG2**	5′-AGUGGUUCUUGCAGUGCUA-3′
**Negative control**	5′-UUCUCCGAACGUGUCACGU-3′

Supplementary Table S2: Primers for real-time qPCR

Gene	Forward primer (5′ to 3′)	Reverse primer (5′ to 3′)	ProductSize (bp)
**CSIG**	CGTATTGGTCACGTTGGAATGC	CCACTTCTCTGGCAATTTTTCTG	94
**MYC**	GCCACGTCTCCACACATCAG	TCTTGGCAGCAGGATAGTCCTT	72
**18S rRNA**	GTAACCCGTTGAACCCCATT	CCATCCAATCGGTAGTAGCG	151
**GAPDH**	CGACCACTTTGTCAAGCTCA	AGGGGTCTACATGGCAACTG	231
